# Codevelopment of an mHealth App With Health Care Providers, Digital Health Experts, Community Partners, and Families for Childhood Obesity Management: Protocol for a Co-Design Process

**DOI:** 10.2196/59238

**Published:** 2025-03-05

**Authors:** Siao Hui Toh, Courtney Davis, Khairunisa Bte Khaider, Zhi Quan Ong, Ethel Jie Kai Lim, Chu Shan Elaine Chew

**Affiliations:** 1 Physiotherapy Department KK Women's and Children's Hospital Singapore Singapore; 2 Health and Social Sciences Cluster Singapore Institute of Technology Singapore Singapore; 3 Adolescent Medicine Service KK Women’s and Children’s Hospital Singapore Singapore; 4 Singhealth Duke-NUS Paediatric Academic Clinical Programme Singapore Singapore; 5 School of Computing, Institute of Operations Research and Analytics National University of Singapore Singapore Singapore; 6 Department of Nutrition and Dietetics KK Women’s and Children’s Hospital Singapore Singapore

**Keywords:** childhood obesity, mHealth, mobile health, co-design, IDEAS framework

## Abstract

**Background:**

Childhood obesity is increasing in Singapore, with most cases persisting into adulthood and leading to poor health outcomes. The current evidence for childhood obesity interventions shows a clear dose-response effect, where effectiveness improves with an increasing number of treatment hours. A minimum threshold of ≥26 hours over a 2- to 12-month period is required to achieve significant outcomes. The Kick Start Move Smart program is the first online community-based multidisciplinary program to treat pediatric obesity in Singapore. It has demonstrated feasibility and acceptability, with 70% of participants completing the recommended ≥26 hours of intervention. Preliminary data show significantly lower BMI and improved quality of life in participants compared to controls. Successful families are positive outliers who developed strategies for health in the context of an obesogenic environment. This positive outlier approach indicates that solutions to challenges that a community faces exist within certain individual members, and these strategies can be generalized and promoted to improve the health of others in the same community. A mobile health (mHealth) app targeting parents is a critical missing link in the currently available interventions to support parental self-management of childhood obesity. Using a combination of behavioral theory and user-centered design approaches is important for designing mHealth apps. One recommended framework is Integrate, Design, Assess, and Share (IDEAS), which aims to facilitate the development of more effective interventions by engaging perspectives from different stakeholders.

**Objective:**

This study aims to (1) describe the co-design protocol of an mHealth app using the IDEAS framework as a low-intensity intervention or as an adjunct to more intensive existing pediatric obesity interventions and (2) assess the usability, acceptability, and engagement of the app by parents.

**Methods:**

A clinician-led co-design approach will be undertaken with a multidisciplinary team using the IDEAS framework. Phase 1 involves stakeholder engagement and the formation of a core committee and a parent advisory board. Phase 2 involves developing the app content through focus group and expert panel discussions. Phase 3 involves developing a prototype app and gathering feedback. Phase 4 involves piloting the minimum viable product by parent users and evaluating its effectiveness through interviews and questionnaires.

**Results:**

In April 2023, a parent advisory board was formed, and stakeholders were engaged as part of phase 1. Phases 2 and 3 were completed in June 2024. Focus group discussions were held with the parent advisory board and stakeholders to identify family strategies and patient-centric outcomes and provide feedback on the app. As of January 2025, the app is complete, and we are now in the middle of data collection from participants. Participants will provide feedback to the research team, and the app will be updated accordingly.

**Conclusions:**

An evidence-based, theory-driven mHealth app developed using a structured design framework can bridge the gap in delivering multidisciplinary care in community settings for families with overweight children.

**International Registered Report Identifier (IRRID):**

DERR1-10.2196/59238

## Introduction

Childhood obesity rates are rising in Singapore, with most cases persisting into adulthood and leading to adverse long-term psychosocial and health effects. A recent systematic review and meta-analysis highlighted the importance of access to multidisciplinary interventions for children with obesity. The current evidence for childhood obesity interventions shows a clear dose-response relationship, where effectiveness improves with an increasing number of treatment hours. A minimum threshold of ≥26 hours over a 2- to 12-month period is required to achieve significant outcomes [1]. The Kick Start Move Smart (KSMS) program is the first online community-based program to treat pediatric obesity in Singapore. The pilot evaluation has demonstrated feasibility and acceptability, with 70% of the participants completing the recommended ≥26 hours of intervention. Preliminary data on the effectiveness of the program show significantly lower BMI scores and improved quality of life in program participants compared to the control group. Families who achieved the recommended treatment hours in the program also demonstrated improvements in their child’s quality of life, health behaviors (such as increased intake of fruits and vegetables), and BMI. Successful families are positive outliers who developed strategies for health in the context of an obesogenic environment. The theoretical underpinning of the positive outlier approach is that solutions to problems a community faces often exist among certain individuals and that these successful strategies can be generalized and promoted to improve the outcomes of others in the same community [2]. Typically, there is a high degree of heterogeneity in response to lifestyle interventions in pediatric obesity treatment; thus, adopting the positive outlier approach can facilitate the best solutions to support families.

An evidence-based, theory-driven mobile health (mHealth) app that targets parents is a critical missing link in currently available interventions to support parental self-management of childhood obesity [3]. While there has been a rapid growth of mHealth apps to support chronic disease management, achieving meaningful health improvements through the use of mHealth apps remains elusive. Combining approaches from behavioral theory and user-centered design to guide intervention development, coupled with rigorous approaches for evaluation and dissemination, is important to achieve the full potential of mHealth apps. Integrate, Design, Assess, and Share (IDEAS) is one recommended framework, which aims to facilitate the development and dissemination of more effective interventions by engaging the perspectives of different stakeholders [4]. This framework builds on design thinking and integrates user insights throughout the stages of development and the inclusion of theory-driven behavioral strategies.

We hypothesize that codevelopment with health care providers, digital health experts, and successful families to create a high-quality mHealth app for childhood obesity can increase its acceptability and engagement from families. First, we aim to co-design an mHealth app with health care providers, digital health experts, community partners, and successful families using the IDEAS framework. The app will be evidence-based and theory-driven and consist of three core components: (1) consolidation of positive outlier strategies and development of patient-centric outcomes for self-monitoring, (2) synthesis of evidence-based guidelines and integration of theory for behavioral strategies, and (3) incorporation of community resources and integration with community programs. The mobile app is designed to be used independently as a low-intensity intervention or as an adjunct to more intensive existing pediatric obesity interventions to increase overall engagement and effectiveness. Second, we aim to assess the usability, acceptability, and engagement of the mHealth app by parents in the pilot testing phase. Specifically, the usability and acceptability of the mHealth app will be assessed using the validated Mobile App Rating Scale (uMARS) [5] and by self-monitoring of patient-centric outcome measures as determined in the primary aim.

## Methods

### Overview

A clinician-led co-design approach will be undertaken with a multidisciplinary team of researchers, parents of children with obesity, community stakeholders, and digital health experts using the IDEAS framework [4]. The experience-based co-design principle will be followed to design the mHealth app, including gathering stakeholders to participate in the design process and developing a set of feasible care paths by sharing their experiences [6]. Based on the IDEAS framework and co-design principles, 4 iterative phases for the prototype mHealth app have been developed, which are summarized in Table 1.

**Table 1:** Phases of the project and their estimated timelines.

Phase	Activities	Duration	Stakeholders (n=21)
1	Stakeholder engagement Forming the parent advisory board	4 months	Multidisciplinary health care providers (10/21, 48%) Parent advisory board (5/21, 24%) Community stakeholders from Sport Singapore and Health Promotion Board (4/21, 19%) Digital health experts and app developers (2/21, 10%)
2	Developing content Patient-centric outcomes that are clinically relevant Successful positive outlier strategies Integration with community resources Implementation of the app in daily lives	6 months	Multidiscpilinary health care providers Parent advisory board Community stakeholders
3	Sharing proposed contents and features based on results from phase 2 and revisions Sharing prototype app	6 months	Multidiscpilinary health care providers Parent advisory board Community stakeholders Digital health experts and app developers
4	Evaluating and refining the MVP^a^ to assess usability and acceptability and improvement of the app functions	9 months	Parents of children with obesity Digital health experts and app developers

^a^MVP: minimum viable product.

### Ethical Considerations

Ethical approval has been sought from the SingHealth Centralized Institutional Review Board (2022/2035).

### Phase 1: Stakeholder Engagement and Formation of the Core Committee and Parent Advisory Board

#### Stakeholder Engagement

The multidisciplinary team of health care providers will consist of pediatricians, dietitians, nurses, physiotherapists, exercise physiologists, psychologists, and social and medical workers with expertise in childhood obesity, who provide their clinical input in the co-design process. Digital health experts and app developers will also advise on the feasibility of the mHealth app’s requirements and involve the vendor at appropriate time points to effectively transform the information into system requirements and design considerations to achieve an optimal design solution. The app’s privacy and security features and compliance with the Personal Data Protection Act will also be considered. These features have emerged as fundamental from both clinical and patient perspectives in an evaluation of an mHealth app [7].

#### Parent Advisory Board to Learn From Positive Outliers

Patient advisory committees have been used in numerous co-design approaches to obtain patient input on a variety of health care processes. This ensures that the posed research questions and selected outcome measures are relevant and important to patients [8]. In this project, we will convene a parent advisory board to inform the mHealth app development. The parent board will consist of at least 5 parents who previously participated in the KSMS pilot study and successfully attended at least 26 hours of the program. The parent advisory board will be part of 2 focus group discussions in phase 2 and 1 focus group discussion in phase 3 of this project.

#### Formation of the Core Committee

Given the networked nature of the project, which includes the multidisciplinary team, community stakeholders, digital health experts, app developers, and a university partner, the core committee is critical for the project’s success. It builds off our 2 years of experience working with multiple stakeholders across institutions for the KSMS program. The core committee will be chaired by the principal investigator with 2 members from the multidisciplinary team, 2 members from community stakeholders, 1 member from the digital health expert team, 1 member from the parent advisory board, 1 member from the partner university, and 1 clinical research coordinator. At defined intervals (intended to be one per quarter and as required), the core committee will meet by teleconference to review the strategic course and resource allocations. The clinical research coordinator, who is also the project coordinator for KSMS, serves as the lead administrator. The core committee is responsible for initiating and reviewing overall project progress and undertaking operational decisions to ensure that the project goals are aligned. This committee plays a vital role in the mHealth app’s successful implementation into clinical practice and in ensuring the project’s sustainability.

### Phase 2: Development of the mHealth App’s Contents

#### Overview

In phase 2, we plan to conduct 2 focus groups with the parent advisory board (5/21, 24%), 1 focus group discussion with community stakeholders (4/21, 19%), and 2 expert panel discussions with the multidisciplinary health care team (10/21, 48%) to develop the 3 main components of the mHealth app, as detailed in the subsequent sections.

#### Consolidating Contextually Tailored Positive Outlier Strategies and Developing Patient-Centric Outcomes for Self-Monitoring

The 2 focus group discussions with the parent advisory board will identify practices that facilitated their engagement in the intervention and change in health behaviors as well as highlight outcomes that matter most to their families in relation to weight management. These successful positive outlier strategies will be generalized and incorporated into the app with the aim of improving other families’ outcomes. Patient-centric outcomes will be incorporated as part of the self-monitored outcome measures for parents. Parents will also provide input on integrating the app into their daily lives. Parents’ detailed input on activity and nutrition suggestions will be used to make these recommendations actionable and tailored contextually (eg, based on time and day of the week), increasing the effectiveness of “push” notifications and suggestions.

#### Synthesizing Evidence-Based Guidelines and Integrating Theory for Behavioral Strategies

The multidisciplinary team of health care providers will consist of pediatricians, dietitians, nurses, physiotherapists, exercise physiologists, psychologists, and social workers trained in pediatric obesity management. Focus groups with the multidisciplinary team will consolidate practices and incorporate evidence-based strategies, guided by expert committee recommendations for pediatric obesity prevention and Singapore’s Integrated 24-Hour Activity Guidelines for Early Childhood (0-6 years) [9]. The multidisciplinary team will also review strategies from parent advisory board focus groups and evidence-based guidelines to select optimal theory-driven behavioral strategies for guiding the mHealth app design [10]. Incorporating motivational interviewing (MI) in health care settings has been shown to improve intervention engagement and commitment to the behavioral change process [11]. MI is a person-centered counseling method that supports individual autonomy and uses collaborative and nonauthoritarian interaction to work toward an individual’s goal.

#### Incorporation of Community Resources and Integration With Community Programs

The focus group with community stakeholders aims to integrate currently available community resources (web-based and physical) that are relevant to the target population, such as community-based sports programs designed for families.

### Phase 3: Development of the Prototype App

The results of phase 2 will be shared with digital health experts and app developers to outline the required app features and guide the development of multiple prototypes. Studies have shown that developing multiple prototypes in parallel (versus sequentially) leads to a superior final prototype [12]. The prototype will be developed in regulated phases before being shared with the parent advisory board, multidisciplinary team, and community stakeholders to gather their feedback through 3 separate focus groups (one for each stakeholder). The feedback obtained will include user experience, visual design, content, and logic (such as a graphical representation of families’ data). Feedback will be gathered from the clinical team on the integration of the app into clinical workflows, its prescription to families, and the visualization of intervention-related data. The digital health experts and app developers will then review the feedback for integration into the prototype app before a final minimum viable product (MVP) is published.

### Phase 4: Piloting the MVP and Evaluation

A fully functional MVP will be developed to focus on details beyond the prototype phase. The MVP will be pilot-tested by 10 parents for 4 weeks to detect usability issues before the final prototype is developed. Parents of children aged 4 to 7 years old with obesity will be recruited from outpatient clinics. A sample size of 10 participants in think-aloud studies has been shown to detect 80% of usability issues [13]. At the start of the pilot testing, think-aloud walkthrough interviews will be conducted. Participants will be instructed to use the app as they would any newly downloaded app, verbalizing their thoughts throughout the process. They will be encouraged to freely explore the app and ask questions to provide insights into their user experiences. Following the walkthrough interviews, participants will be asked to use the app for 4 weeks.

At the end of the 4-week period, semistructured interviews and the uMARS questionnaire will be administered. The interviews will consist of open-ended questions on participants’ previous experience with mobile apps, general perceptions of the app, its most/least useful features, suggestions for improvement and usage, possible features that could exclude potential users, and factors related to engagement*.* Inputs on the push notifications will be obtained from parents to optimize their timing and delivery to target behavior change.

Analytics will be integrated into the app to collect a rich dataset, capturing patterns of use, particularly in relation to accessing information and completing patient-centric outcomes as defined in our secondary aims. The usability and acceptability of the mHealth app’s “pull” features (educational materials and graphical representations of monitored outcomes) will be evaluated using uMARS, a 20-item measure that includes 4 objective quality subscales (engagement, functionality, aesthetics, and information quality), 1 subjective quality subscale, and 1 perceived impact scale, all measured on a 5-point Likert scale. A score of 4 and above indicates good quality [5].

High usability and acceptability will be defined as a median score of 4 out of 5 in all domains (information, engagement, functionality, aesthetics, impact, and acceptability) for parents. The user engagement aims will be defined as (1) recruited parents accessing ≥70% of the information and (2) completing patient-reported outcome measures over the 4-week period.

## Results

This study received funding in February 2023. As part of phase 1, a parent advisory board was formed in March 2023 with parents who had previously participated in the KSMS pilot study and completed at least 26 hours of the program. Stakeholders were engaged in April 2023 to co-develop the mHealth app. Stakeholders included the multidisciplinary health care team from KK Women’s and Children’s Hospital, the Health Promotion Board (HPB), and Sport Singapore. Digital health experts and app developers from the National University of Singapore School of Computing were also involved in developing the mHealth app.

Phase 2 was completed between April 2023 and December 2023. The first focus group discussion with the parent advisory board was held in April 2023 to identify positive outlier strategies. A preliminary mock-up app was developed and shared in focus group discussions for feedback with community stakeholders (HPB and Sport Singapore) in May 2023, followed by the multidisciplinary team in July 2023. Discussions were held with community stakeholders between June 2023 and December 2023 to integrate existing community resources and programs into the mHealth app. A second round of individual interviews with the parent advisory board was conducted in October 2023 to identify patient-centric outcomes and review the mock-up app.

Revisions were made to the mHealth app following feedback from the stakeholders and parent advisory board. Focus group discussions and individual interviews were conducted in phase 3 to obtain feedback on the revised mock-up app with stakeholders and the parent advisory board, completed between April and June 2024.

The final stage of the mHealth app development was completed in December 2024 with an MVP app. Participants were recruited to pilot-test the mHealth app and provide feedback from January 2025. Recruitment will resume in April 2025, and feedback will be collected from the participants to enhance the mHealth app.

## Discussion

### Expected Findings

This study outlines the co-design process of an mHealth app with health care providers, digital health experts, community partners, and families, using the IDEAS framework. We hypothesize that the app, developed through the IDEAS framework, will be usable, acceptable, and engaging for parents in managing childhood obesity.

Patient advisory committees have become increasingly important in health care, offering valuable insights into various health conditions [14]. Engaging parents as active participants in the design of this app is important to ensure that the strategies recommended for other parents will be relevant and meaningful. For patient-centric outcomes, we anticipate focusing on themes related to psychosocial wellness, as seen in other studies [15], especially considering the lower quality of life among Singaporean children with obesity [16].

Some successful positive outlier strategies identified in a United States–based study [15] include (1) making family-centric changes in health behaviors rather than child-centric changes, (2) establishing rules around health behaviors, (3) shared decision-making with health care providers on child weight management, and (4) using community resources for behavioral change. We anticipate some common themes among our local population, especially around family-centric changes, rule implementation on behavior change, and the use of immediate outcomes, which align with findings from our previous study [17]. However, strategies like shared decision-making with health care providers and the use of community resources are less likely to emerge as important themes. This is because obesity is commonly perceived as a lifestyle factor, rather than a chronic disease, with treatment not claimable under private or public health care insurance [18]. These factors may hinder help-seeking for obesity treatment and pose challenges in developing community programs for childhood obesity management. Thus, the development of this mHealth app aims to bridge the gap in available resources for managing childhood obesity.

Despite research showing that parents are an integral part of pediatric obesity prevention and treatment, the currently available apps do not target parents or families but focus solely on the child. A review of mHealth apps for pediatric obesity prevention and treatment also found that most apps lacked any expert recommendations. mHealth apps have also been found to lack a theoretical basis for their development, thereby limiting their efficacy [19]. There is currently no mHealth app that integrates the strategies learned from positive outlier families with locally available community resources. Thus, this project will be the first theory- and evidence-informed mHealth app for pediatric obesity management targeted toward parents. The successful development of this mHealth app, through the use of the IDEAS framework, will also provide important information for the future development of mHealth apps for other chronic diseases, such as diabetes mellitus, to facilitate behavioral change and improve patient outcomes.

Currently, primary care providers and patients can only access pediatricians, dietitians, physiotherapists, and exercise physiologists trained in pediatric obesity management when they are referred to tertiary pediatric centers. This reliance on specialized centers can result in these services becoming overwhelmed. An mHealth app that is evidence-based, theory-driven, and developed within a design framework, can bridge this gap in providing multidisciplinary care in the community setting. Health care providers in both tertiary and primary care often face significant time constraints during consultations. Engaging these health care providers in the app’s development and evaluation process can enhance its usability and acceptability, enabling its incorporation into clinical care processes.

The proposed mHealth app will thus provide the foundation for accessible multidisciplinary care in the community, scaling up the KSMS program to provide evidence-based treatment to every family with overweight children. This approach aims to help reduce complications associated with obesity.

**Figure 1:**
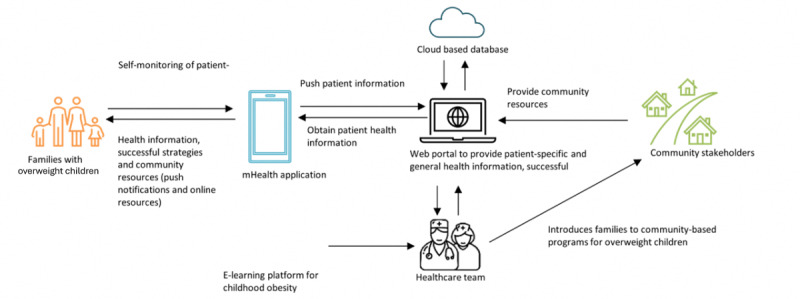
Proposed mechanism of the mobile health (mHealth) app and its future integration into health care settings, health care provider training, and community programs.

By evaluating the potential effectiveness of these multimodal complex interventions (Figure 1) in reducing childhood obesity, improving quality of life, and reducing health care visits, this study could provide cost-effectiveness data to support informed decisions for nationwide implementation.

### Strengths and Limitations

The key strengths of this study include the co-design approach with a multidisciplinary team and parents, as well as the development of an evidence-based, theory-driven mHealth app. These elements are critical for ensuring effective digital interventions that promote behavior change in childhood obesity management.

However, this study has limitations. Nonresponder bias may arise, as responses from participants not identified as positive outliers are not captured. Additionally, the effectiveness of the mHealth app compared to standard care is being assessed in this study.

### Conclusions

An evidence-based, theory-driven mHealth app developed within a structured design framework can bridge the gap in providing multidisciplinary care for families with overweight children in the community setting. This app will also empower families to improve self-management of childhood obesity. Future studies are required to examine the effectiveness of this mHealth app in managing childhood obesity and promoting better self-management compared to standard care.

## Appendix

Multimedia Appendix 1 Peer-review report by the Human Potential Prenatal / Early Childhood Grant Office Agency for Science, Technology, and Research (Singapore).

## Abbreviations

HPB: Health Promotion Board
IDEAS: Integrate, Design, Assess, and Share
KSMS: Kick Start Move Smart
mHealth: mobile health
MI: motivational interviewing
MVP: minimum viable product
uMARS: Mobile App Rating Scale

## Data Availability

Data sharing is not applicable to this article as no datasets have been generated or analyzed during this study.
