# Supplementary material for: Codevelopment of an mHealth App With Health Care Providers, Digital Health Experts, Community Partners, and Families for Childhood Obesity Management: Protocol for a Co-Design Process
Source: JMIR Res Protoc. 2025 Mar 5;14:e59238. doi: 10.2196/59238 (PMC11923461; doi:10.2196/59238)
Supplement: Multimedia Appendix 1 [file resprot_v14i1e59238_app1.pdf]

Agency for Science, Technology and Research
SINGAPORE

Biomedical Research Council
(BMRC)
20 Biopolis Way
#07-01, Centros
Singapore 138668

12 Jul 2024

By Email

Dr Elaine Chew Chu Shan
Senior Consultant
KK Women's and Children's Hospital

Dear Dr Chew,

As per your request, please find a summary of the comments from our reviewers for the proposal you submitted during the FY21 Prenatal/Early Childhood Grant Call.

Proposal ID: PEC21-0061 (H22P0M0010)
Project Title: Co-development of a mobile health application with healthcare providers, digital health experts and successful families for childhood obesity
Lead PI: Dr Elaine Chew Chu Shan

Summary of Comments	Key Strengths/Weaknesses
The proposal addresses a significant problem via a program that Strengths: has shown good feasibility, acceptability and potential efficacy in the pilot phase. The proposed investigation process is also clear and comprehensive. The reviewers were positive with the feasibility and acceptability data generated from the pilot program as the use of online community approach and the user- centered design could enhance the effectiveness of the study. Also, the suggested framework appears highly appropriate for the project development and implementation. While the idea is original, innovative, strong and well justified, the reviewers were concerned with the validity of the pilot data (small sample size and lack of publication), and the uncertainty of the take-up rate. There was also concern about the fundamental theory that will be used to design the intervention, assessment of the outcomes and its effectiveness. Nonetheless, the proposal has strong potential for deployment with a well described plan for community engagement, app development and initial feasibility testing.	1. Highly experienced research team<br>2. Leverage on the subjects involved in the pilot study 3. Great potential for scaling up 4. Involvement of stakeholders<br><br>Weaknesses:<br>1. Unclear validity of findings in pilot study<br>2. Lack of clarity in the utility, engagement and support for the execution platform (Singhealth Health Buddy) as a behaviour changing tool<br>3. Lack of details of the outcomes of the project

Thank you and we wish you all the best for the manuscript submission.

Yours Sincerely,

xxxxxx
